# From Waste to Resource: Exploring Green Approaches for Phenolics Recovery from Olive Leaves

**DOI:** 10.3390/antiox14020136

**Published:** 2025-01-24

**Authors:** Paulina Tapia-Quirós, Aina Mir-Cerdà, Mercè Granados, Sonia Sentellas, Javier Saurina

**Affiliations:** 1 Department of Chemical Engineering and Analytical Chemistry, Universitat de Barcelona, Martí i Franquès 1-11, E08028 Barcelona, Spain; paulina.tapia@ub.edu (P.T.-Q.); ainamir@ub.edu (A.M.-C.); mgranados@ub.edu (M.G.); sonia.sentellas@ub.edu (S.S.); 2 Chemical Engineering Department, Escola d’Enginyeria de Barcelona Est (EEBE), Universitat Politècnica de Catalunya (UPC)-BarcelonaTECH, Eduard Maristany 10-14, Campus Diagonal Besòs, E08930 Barcelona, Spain; 3 Research Institute in Food Nutrition and Food Safety, Universitat de Barcelona, Av. Prat de la Riba 171, Edifici Recerca (Gaudí), E08921 Santa Coloma de Gramenet, Spain; 4 Serra Húnter Fellow, Generalitat de Catalunya, E08003 Barcelona, Spain

**Keywords:** microwave-assisted extraction, phenolic compounds, natural deep eutectic solvents, agri-food waste valorization, circular economy

## Abstract

Waste management presents a significant challenge for agri-food industries, but also an opportunity to recover valuable bioactive compounds, particularly phenolics, aligning with circular economy principles. This study compares the efficacy of conventional solvents and natural deep eutectic solvents (NADES) in extracting polyphenols from olive leaves using the scalable techniques of solid–liquid extraction (SLE) with mechanical stirring and microwave-assisted extraction (MAE). Key factors affecting extraction yield, including solvent composition, time, and temperature were investigated. Extraction efficiency was evaluated by measuring total polyphenol content (TPC) by high-performance liquid chromatography (HPLC), antioxidant capacity (FRAP assay), and individual phenolic compounds, also quantified using HPLC. Additionally, differential pulse voltammetry (DPV) was employed to evaluate the antioxidant quality of the extracts. NADES demonstrated superior extraction performance compared to conventional solvents, with the choline–glycerol system showing the highest efficiency. The combination of MAE and NADES emerged as a green and sustainable alternative to traditional methods, offering improved yield and speed. In contrast, SLE with water or ethanol/water mixtures required extended times or higher temperatures for comparable results but offered easier scalability for industrial applications.

## 1. Introduction

Olive oil production is a major economic activity in Southern Europe. It is estimated that 2.55 million metric tons of olive oil are produced per year. Therefore, this industry generates large amounts of solid and liquid waste, about 10 million tons of waste per year, including olive pomace, wastewater, and olive leaves as the main ones [1]. Leaves from *Olea europaea* contain a higher quantity of phenolic compounds compared to fruit or virgin olive oil [2] (ca. 1450 mg of total phenols/100 g of fresh leaves [3] in contrast to 110 mg/100 g of fruit [4], and 23 mg/100 mL of extra virgin olive oil [2,5]).

Phenolic compounds are an extensive group of secondary plant metabolites, synthesized as protective agents against external environmental or parasitic aggressions, with powerful activities as antioxidant and antiradical agents to scavenge free radicals, as structural molecules for the formation of protective parts in plants against insects, or as antibiotics to combat microorganisms that can affect the plant [6,7,8]. Under the generic framework of polyphenols, various families of compounds widespread throughout the plant kingdom, such as phenolic acids and flavonoids, are included [9,10]. However, phenylethanoids also stand out in olives as abundant and strategically remarkable chemicals. In general, phenolics arouse special interest in the food, cosmetic, and pharmaceutical industries because of their noticeable attributes and benefits for human health [8]. Olive leaves have been used from ancient times in traditional medicine to make infusions for a wide range of ailments [11,12]. Olive leaves contain a large number of phenolic compounds such as oleuropein, tyrosol, hydroxytyrosol, luteolin, coumaric acid, caffeic acid, ferulic acid, vanillic acid, ligstroside, rutin glucosides, etc. [13,14,15,16,17]. In recent times, there has been scientific evidence about their potential health benefits, with anti-inflammatory, anticarcinogenic, antihypertensive, antiviral, antimicrobial, hypoglycemic, antithrombotic, and hypocholesterolemic effects [11,12].

Phenolic compounds are commonly extracted with organic solvents, such as methanol, acetone, or ethyl acetate [18,19,20,21]. Nevertheless, these solvents are toxic and unsuitable (even incompatible) for food, cosmetic, and pharmaceutical purposes. For this type of application, the most appropriate solvent is water, but the extraction yields may be lower than other options, especially for apolar compounds such as flavonoids and phenylethanoid derivatives (e.g., oleuropein, with apolar moieties in their structures). Ethanol is an excellent option to improve the analyte affinity towards the solvent without incurring incompatibilities with food or nutraceutical applications, especially when used in hydro-organic mixtures.

Natural deep eutectic solvents (NADES) provide a green alternative to conventional solvents because of their enhanced extraction performance [18,22,23,24]. NADES consist of a mixture of two or more components, a hydrogen bond acceptor (e.g., choline chloride) and a hydrogen bond donor (e.g., glycerol), at a particular molar ratio to formulate eutectic mixtures. NADES have distinctive physicochemical properties, such as adjustable viscosity, low volatility, water solubility, strong solubilization capability for a wide range of compounds, and pharmaceutically acceptable toxicity [18,22,23,24].

Several extraction techniques have been proposed to recover polyphenols from olive mill waste, ranging from solid–liquid extraction (SLE) by simple mechanical stirring to advanced techniques (e.g., ultrasound-assisted extraction (UAE), pressurized liquid extraction (PLE), or microwave-assisted extraction (MAE)) [25,26]. SLE is the most widely used technique for the extraction of polyphenols from agri-food residues due to its ease of implementation and reduced operating costs. Beyond SLE, which is readily scalable for pilot plant or industrial applications, UAE and MAE are also implementable with reasonable ease. In particular, MAE proves to be a highly efficient technique, providing high extraction yields in a short processing time. Microwave radiation causes cell walls in the waste matrix to swell and rupture, which enhances the release of target compounds [24,27]. Additionally, MAE offers simplified handling, low energy requirements, and reduced solvent consumption.

This paper addresses the recovery of bioactive phenolics from the olive leaf matrix using scalable extraction techniques and green solvents, including some conventional solvent mixtures and NADES. The study evaluates the extraction capacity of different techniques and solvents to identify the most appropriate approach based on diverse outcomes such as the antioxidant capacity estimated by the ferric reducing antioxidant power (FRAP) assay, the global phenolic content determined from the chromatographic profile recorded at 280 nm, and the concentration of quantitatively remarkable compounds, such as luteolin, hydroxytyrosol, and their derivatives. Additionally, an electrochemical study of the antioxidant properties of the extract has been carried out to establish the compounds with the greatest reducing capacity, i.e., those that are easily oxidized at lower potentials and are expected to be the most powerful or proactive.

## 2. Materials and Methods

### 2.1. Chemicals

Polyphenol standards were purchased from the following suppliers: gallic acid, *p*-coumaric acid, oleoside, luteolin, and luteolin-7-O-glucoside were from Sigma Aldrich (St. Louis, MO, USA); rutin and 3-hydroxytyrosol were from TCI (Tokyo, Japan); oleuropein and oleuropein aglycone were from Biosynth Carbosynth (Berkshire, UK). Stock solutions of each of the compounds were prepared in methanol at a concentration of ca. 5000 mg L^−1^ and were stored in the freezer at −20 °C until use. The standard solutions for calibration were prepared from these stock solutions.

Reagents used for spectrophotometric assays were: 2,4,6-tripyridyl-S-triazine (TPTZ) from Alfa Aesar (Kandel, Germany), Fe(III) chloride from Merck (Darmstadt, Germany), Trolox from Carbosynth (Berkshire, UK).

Chemicals for the preparation of extraction solvents and the mobile phase were as follows: choline chloride (ChCl), glycerol (Gly), and urea from Thermo Fisher (Kandel, Germany); lactic acid (Lac) from Acros Organics (Geel, Belgium); ethylene glycol (EG) from PanReac (Barcelona, Spain); ethanol (EtOH) from Honeywell, Riedel-de Haën™ (Offenbach, Germany); acetonitrile (ACN) from Fisher Scientific (Leicestershire, UK); dimethylsulfoxide (DMSO), formic acid (FA) 98–100% *w*/*w*, and hydrochloric acid (HCl) 32% *w*/*w* from Merck (Darmstradt, Germany). Ultrapure water was generated with a Milli-Q system (Merck Millipore, Bedford, MA, USA).

### 2.2. Samples and Sample Treatment

Leaves from various olive tree (*Olea europaea* L.) cultivars, such as Arbequina and Verdiella, were obtained during extra virgin olive oil production in November 2021 in Albelda (Huesca, Spain). The olive harvest was sifted to separate the leaves from the fruit, and the leaf samples were then collected. The fresh leaves were processed in the laboratory with a domestic grinder for 1 min and then sieved through a 2 mm mesh. The resulting powder was stored at −18 °C until being used.

The samples were extracted with various solvents and techniques to evaluate the effectiveness of each procedure in the recovery of phenolic compounds. Extracts were filtered with nylon syringe filters (13 mm, 0.45 μm) from Agilent Technologies (Waldbronn, Germany). All conditions were tested in duplicate to have information on the variability of the results and their statistical significance.

### 2.3. NADES Preparation

Several NADES compositions based on ChCl and various donors, such as Gly, urea, Lac, and EG (see Section 2.1), were studied to extract phenolic compounds. The acceptor and donor components were mixed in a molar ratio of 1:5 and heated with constant stirring at 60 °C in a water bath until a colorless and homogeneous liquid was obtained. Then, 30% water was added and mixed [26]. Also, conventional solvents like ethanol/water solutions and water were compared.

### 2.4. Extraction Procedures

#### 2.4.1. Microwave-Assisted Extraction (MAE)

The Milestone Microwave Labstation system (Ethos E, Milestone, Shelton, CT, USA) was used for MAE with NADES. The procedure used one gram of fresh sample placed in a PTFE vessel with 20 mL of the corresponding NADES system. Stirring was set at 50% with a magnet inside each vessel and power at 500 W. The influence of solvent composition, temperature (80–180 °C), and time (5–15 min) on the phenolic recovery was assessed by a factorial experimental design. After extraction, samples were transferred to a Falcon tube, centrifuged for 15 min at 3500 rpm with the Labofuge 400 centrifuge, filtered with nylon syringe filters, and stored at 4 °C until analysis. Experiments were performed in duplicate.

#### 2.4.2. Solid–Liquid Extraction (SLE)

SLE with NADES and conventional solvents relied on a previously optimized methodology [26] using an RCT basic hot plate stirrer with temperature controller (IKA^®^, Staufen, Germany). Briefly, 1 g of fresh sample was mixed with 20 mL of the corresponding solvent. The extraction was at 80 °C in a water bath with constant stirring using a magnet (450 rpm) with the extraction time varying from 5 min to 2 h. After each batch, the extracts were centrifuged for 15 min at 3500 rpm with a Labofuge 400 centrifuge (Heraeus, Hanau, Germany), filtered with nylon syringe filters (13 mm, 0.45 μm; Agilent Technologies, Waldbronn, Germany), and stored at 4 °C until analysis. Experiments were performed in duplicate.

### 2.5. Analysis Methods

#### 2.5.1. Ferric Reducing Antioxidant Power Assay (FRAP)

The antioxidant activity of samples was estimated spectrophotometrically by the FRAP method adapted from the publication by Alcalde et al. [27] using an Agilent 8453 UV-Vis spectrophotometer (Santa Clara, CA, USA) with QS quartz glass high-performance cuvettes (10 mm optical path) from Hellma Analytics (Jena, Germany). The FRAP reagent consisted of 20 mmol L^−1^ FeCl3 solution, 10 mmol L^−1^ TPTZ solution (50 mmol L^−1^ HCl), and 50 mmol L^−1^ FA buffer solution, mixed in a 1:2:10 (*v*/*v*/*v*) proportion. A volume of 300 μL of FRAP was mixed with appropriate sample volumes and diluted with Milli-Q water to a final volume of 2.5 mL. The reaction was developed for 5 min, and the absorbance was measured at 595 nm using a reagent blank as the reference. Trolox standards in the concentration range of 0.2 to 10 mg L^−1^ were used for calibration. Antioxidant capacity was expressed as Trolox equivalent units per gram of fresh sample (mg TE g^−1^). Analyses were performed in duplicate.

#### 2.5.2. Liquid Chromatography with UV Detection (HPLC-UV)

HPLC-UV was used to determine the global content of phenolic compounds and quantify relevant compounds such as luteolin glucoside, luteolin, *p*-coumaric acid, or rutin using method conditions optimized elsewhere [26]. The instruments used consisted of an Agilent Series 1200 system (Agilent Technologies, Palo Alto, CA, USA) equipped with a quaternary pump, an automatic injection system, and a diode array detector (DAD). Phenolic compound separated under the reversed-phase mode with a Kinetex C18 column (100 mm × 4.6 mm × 2.6 µm, Phenomenex, Torrance, CA, USA) using 0.1% formic acid aqueous solution (A) and ACN (B) as the mobile phase components. The gradient program was as follows: 0 min, 3%B; 0–18 min, 30%B; 18–28 min, 65%B; 28–30 min, 90%B; 30–32.5 min, 90%B; 32.5–33 min, 3%B; 33–40 min, 3%B. The flow rate was 0.5 mL min^−1^ and the injection volume was 10 µL. Chromatograms were recorded at 280, 325, and 370 nm. The total polyphenol content (TPC) was estimated from the total peak area at 280 nm, in the time range between 5 and 30 min. TPC was expressed in terms of mg of gallic acid equivalent (GAE) per gram of fresh sample (mg GAE g^−1^). Analogously, specific phenolics were determined from the corresponding peak area at suitable wavelengths of 280 nm for 3-hydroxytyrosol, oleoside, oleuropein, and oleuropein aglycone; 325 nm for *p*-coumaric acid; and 370 nm for luteolin, luteolin-7-O-glucoside, and rutin. Calibration curves were built for each analyte using the corresponding standards, in the concentration range of 0.5 to 20 mg L^−1^.

#### 2.5.3. Differential Pulse Voltammetry (DPV)

The redox features of the most significant compounds and the ethanolic or NADES extracts were evaluated electrochemically with a µAutolab system Type (III) instrument (EcoChemie, Utrech, Netherlands) attached to a 663VA Stand (Metrohm, Herisau, Switzerland), using a screen-printed carbon DRP-110 device (Dropsens, Oviedo, Spain) as the working, reference, and auxiliary electrode. DPV was used for measurements according to the conditions by Alcalde et al. [27]. Briefly, an appropriate volume of sample or standard was added to 10 mL of supporting electrolyte (0.1 M sodium acetate–acetic acid buffer at pH 5) in the electrochemical cell. Differential pulse voltammograms were recorded from −0.5 to +0.1 V. Other conditions were scan rate 0.25 mV s^−1^, potential step 10 mV, pulse potential 100 mV, and pulse time 0.01 s. Data was acquired with the GPES 4.9 software.

#### 2.5.4. Statistical Analysis

The significance of factors was evaluated by ANOVA and Students’ *t*-test using Microsoft Excel 2016. The significance level was 0.05.

#### 2.5.5. AGREE Score Calculation

The AGREEprep calculator [28], based on the principles of green chemistry, was used to assign Analytical Greenness Metric (AGREE) scores to the extraction procedures.

## 3. Results and Discussion

This paper evaluates the effectiveness of phenolic compound extraction from olive leaves, considered a waste matrix from the extra virgin oil production process. The extraction capacity of various green solvents was compared, including water, ethanol/water solutions, and NADES formulated with ChCl as acceptor and donor agents such as Gly, EG, urea, or Lac. These donors have demonstrated their efficiency in other similar cases [26].

**Figure 1:**
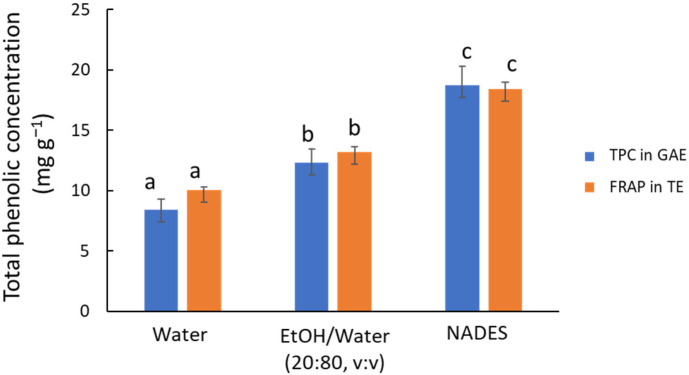
Recovery of phenolic compounds from olive leaves using water, ethanol/water mixture (20:80 *v*/*v*), and ChCl/Gly NADES (1:5 m/m, 30% water) using MAE as an extraction technique. Blue bars, TPC values by HPLC-UV in mg GAE g^−1^; orange bars, antioxidant capacity values by FRAP in mg TE g^−1^. Comparing TPC or TE values, different letters mean significant differences and vice versa.

The first preliminary study aimed to compare three green solvent systems using MAE (10 min at 80 °C) as the extraction technique, as follows: water, a 20:80 (*v*/*v*) ethanol/water solution, and ChCl/Gly. The FRAP assay reveals significant differences (*p* < 0.001) in antioxidant capacity among the three solvents (see orange bars in Figure 1), with the NADES demonstrating superior performance compared to the other solvents. This result agrees with previous studies by other authors who compared NADES with conventional organic solvents and water [2,22]. Water is less effective for extracting less polar compounds, with the antioxidant capacity of the aqueous extract being 65% lower than that of the NADES extract. However, water-based systems remain a viable option for industrial applications due to their environmental benefits and low cost. Adding 20% ethanol, a conventional green organic solvent, increases extraction efficiency by 1.5 times without considerably raising costs. In line with this, Jurmanović et al. [29] proposed a 20:80 (*v*/*v*) ethanol/water mixture for extracting polyphenols from olive pomace using MAE.

The TPC results determined by HPLC (see blue bars in Figure 1) led to similar conclusions, indicating that NADES was the most efficient option (*p* < 0.001). The concentration of individual compounds in the extracts (Figure S1) provided a more detailed picture of the behavior of the different extraction media. In particular, flavonoid glycosides (luteolin glucoside and rutin) and hydroxycinnamic acids (*p*-coumaric acid) follow a pattern similar to that set out above, but aglycones (e.g., luteolin) are hardly extracted by water or the hydroalcoholic mixture because of their lower polarity.

The extraction performance of conventional solvents can be significantly improved when working at higher temperatures, reaching yields comparable to NADES. For instance, in the MAE assay for 10 min at temperatures ranging from 80 °C to 180 °C (Figure S2), ANOVA showed no significant differences in FRAP activity between ethanol/water (20:80 *v*/*v*) and ChCl/Gly. With water at 80 °C, the FRAP index was considerably lower than for the other systems; it increased progressively with temperature (up to 3.8 times above 160 °C, reaching ca. 85% of the antioxidant capacity achieved by the other solvents). In the literature, various authors proposed MAE extraction temperatures of 70–80 °C for olive leaves using NADES [2,18,22].

**Figure 2:**
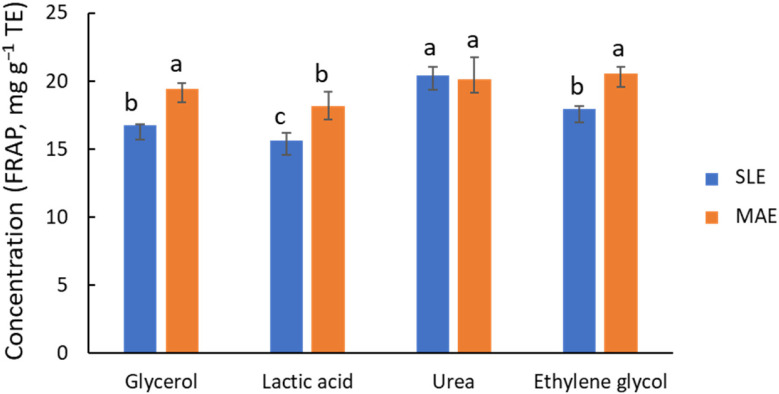
Influence of NADES composition on the recovery of phenolic compounds, estimated by FRAP, from olive leaves using MAE (orange bars) and SLE (blue bars) as extraction techniques. Different letters mean significant differences and vice versa.

Based on their promising performance in similar studies [2,22], the extraction efficiency of four NADES, composed of ChCl with Gly, Lac, urea, or EG as donors, was compared using a 1:5 ChCl-to-donor ratio and 30% water. In addition to the MAE technique (10 min at 80 °C), conventional solid–liquid extraction (SLE) with mechanical stirring (2 h at 80 °C) was also considered. The outcomes of global antioxidant capacity by FRAP (Figure 2) indicate that Gly, EG, and urea provide high activities, ca. 15 to 20 mg g^−1^ TE, with urea possibly being the most effective donor. In contrast, Lac was found to be a less effective agent. The behavior of Gly and EG is similar, in line with the structural and functional similarity of these two molecules. Except for the ChCl/urea system, Figure 2 shows that MAE provides FRAP values for global antioxidant capacity that are ca. 20% higher than those of SLE. However, there are noticeable differences in process time (2 h for SLE vs. 10 min for MAE). It is important to note that shorter process times in SLE provided significantly lower extraction yields (Figure S3). Chanioti et al. [24,30] compared MAE, SLE, UAE, and high hydrostatic pressure extraction, and found that SLE provided the highest extraction efficiency for recovering phenolic compounds from olive pomace matrix. In that study, the authors selected ChCl/citric acid (1:2) with 20% of water or ChCl/caffeic acid (1:2), 60 °C, and 30 min of extraction time as the working conditions.

When examining the chromatographic results (Figure S4), the behaviors of luteolin glycoside and rutin under the different conditions are similar, with slightly higher values observed in the case of SLE and NADES containing urea. Luteolin results are more erratic, likely due to potential degradation processes. Overall, TPC is ca. 20 mg g^−1^ GAE in the extracts for both MAE and SLE, highlighting the potential of this matrix as a source of these phytochemicals.

**Figure 3:**
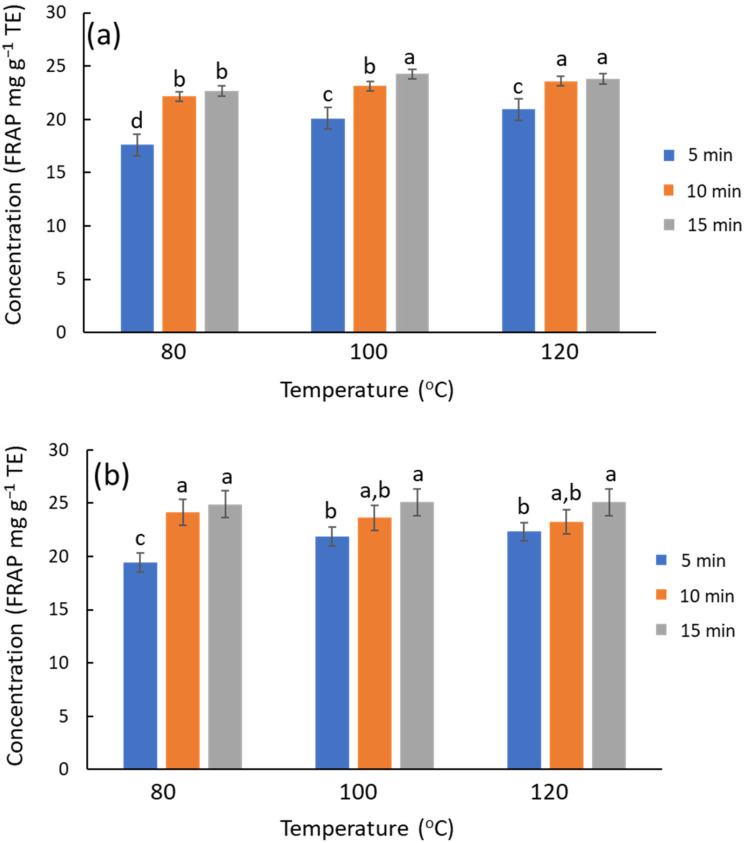
Influence of time and temperature on the recovery of phenolic compounds from olive leaves using MAE, estimated by FRAP. (**a**) ChCl/Gly, (**b**) ChCl/urea. For each NADES system, different letters mean significant differences and vice versa.

The extraction performance of MAE was assessed with more detail by an experimental design of two factors at three levels: time (5, 10, and 15 min) and temperature (80, 100, 120 °C). NADES systems formulated with Gly and urea were chosen as representative cases. The trends are similar in both systems, and the antioxidant capacity of urea and Gly extracts is similar (on average ca. 5% higher for urea) (Figure 3). The significance of extraction time and temperature factors was evaluated by ANOVA using antioxidant activity and TPC as the data. The antioxidant capacity from FRAP (Figure 3a) showed that increasing temperature or time slightly improved the antioxidant capacity of the extracts for the ChCl/Gly system (temperature, *p*-value = 0.0031; time, *p*-value = 0.035), while the interaction was irrelevant. Similarly, these factors were also relevant for the urea-based NADES (temperature, *p*-value = 3.4 × 10^−6^; time, *p*-value = 5.8 × 10^−10^; interaction, *p*-value = 0.0015) The chromatographic results lead to similar conclusions on the influence of temperature and time on the TPC; the response increases with these factors, although the effect of temperature is quantitatively more notable. ChCl/urea outcomes (Figure S5) were ca. 20% higher than ChCl/Gly ones (Figure S6), with the differences becoming accentuated at elevated temperatures. However, chromatographic profiles denoted that these differences are not attributable to polyphenols but rather to degradation products associated with urea that interfered with the chromatograms; this process contributes to the overall response, though the antioxidant capacity of these degradation products is null. Therefore, urea-based NADES should be avoided under more rigorous conditions, e.g., high temperatures or prolonged processing times, as this decomposition also entails an unpleasant odor. Consequently, urea-based NADES are not recommended for applications in food, nutraceuticals, or cosmetics. Besides, when focusing on given polyphenols (luteolin glucoside, rutin, and luteolin), the extraction improves slightly with temperature or time (Figure S6), but the results with urea are not superior to those with glycerol.

**Figure 4:**
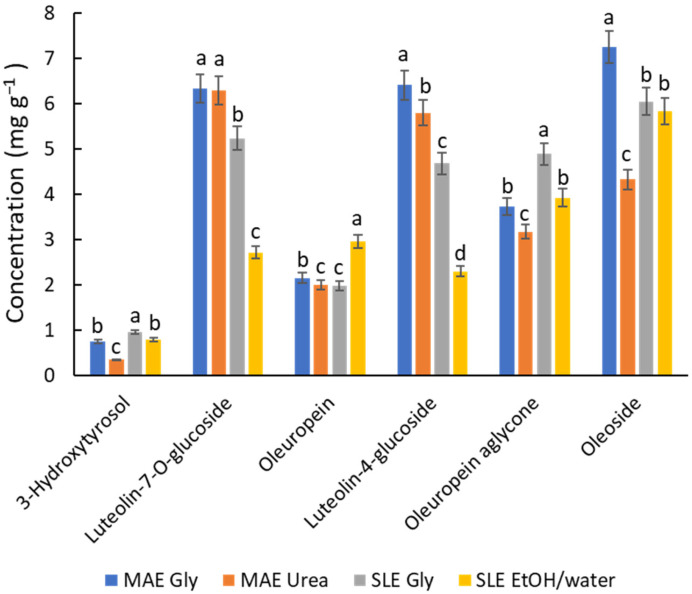
Recovery of the main phenolic compounds, expressed in mg g^−1^, determined by HPLC depending on the solvents and extraction techniques under optimal conditions. For each compound, different letters mean significant differences and vice versa.

In light of all the previous results, a final study was carried out to compare the options considered most relevant for various reasons. The selected conditions include MAE (80 °C, 10 min) with Gly and urea NADES, and SLE (80 °C, 2 h) with Gly NADES and an ethanol/water mixture. Figure 4 shows the profiling of the most quantitatively significant polyphenols, such as 3-hydroxytyrosol, luteolin-7-O-glucoside, oleuropein, luteolin-4-glucoside, oleuropein aglycone, and oleoside, which were found at concentrations above 1 mg g^−1^ in the sample, representing ca. 90% of the total polyphenols. On the whole, the MAE/NADES Gly system provides better recoveries for flavonoids; this finding is in accordance with the results of Gao et al. [31] dealing with the flavonoid extraction (rutin, isoquercetin, astragalin) from Mulberry leaves using MAE (ChCl/Gly (1:2) with 20% of water). When comparing the performance of SLE and MAE (Figure 4), MAE is generally superior. However, SLE with either ChCl/Gly NADES or the hydroalcoholic mixture (20% EtOH) is more suitable for hydroxytyrosol derivatives (e.g., 3-hydroxytyrosol and oleuropein). This outcome is in line with Boli et al. [18], who showed that ethanol is an effective solvent for oleuropein recovery from olive leaves by SLE, particularly at high temperatures (70 °C). For compounds occurring at concentrations below 0.5 mg g^−1^, such as rutin, coumaric acid, and luteolin, the general behavior seems more complex, and their extraction yield may depend on unknown affinity issues. Overall, since the quantitative differences between the options are not remarkable, it suggests that all approaches are viable. The choice between them will depend on the specific applications of the extracts and the scaling requirements.

**Figure 5:**
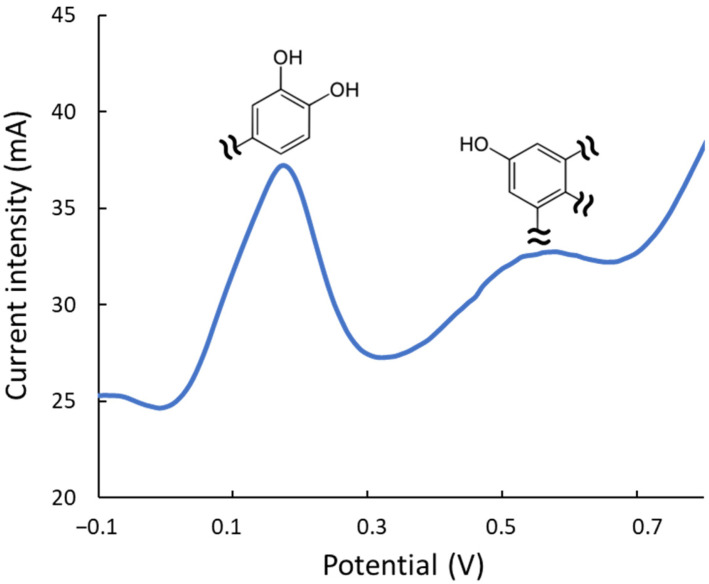
DPV of an ethanolic extract (ethanol/water 20:80, *v*/*v*) of olive leaves with the peaks of the two main types of antioxidant moieties.

Electrochemical studies were also performed to estimate the antioxidant quality of the extracts. On the one hand, the intensity of the DPV signals is a quantitative measurement of the overall antioxidant rate, i.e., providing analogous information to the FRAP or TPC index. On the other hand, the reduction potential provides a qualitative idea of the potency to neutralize or combat different types of oxidizing compounds in the medium. Hence, DPV peaks at lower potentials correspond to more active reducing extracts/compounds as excellent protectors against oxidation processes. Figure 5 depicts a representative voltammogram of an ethanol/water extract. As mentioned, the peak at 0.2 V corresponds to the most electroactive compounds, such as hydroxytyrosol, luteolin, and their derivatives, which contain dihydroxyphenyl moieties (hydroxyphenyls with OH- groups in ortho- or para-positions). The band at 0.6 V is associated with less active phenolic groups, including moieties with simple hydroxyphenyl or dihydroxyphenyls with OH- groups in meta. DPV analysis showed the Gly-based extracts are 20% more active than the urea ones. The effect of the extraction conditions on the antioxidant quality was also confirmed, observing that when working at elevated temperatures, the most labile compounds (peak at ca. 0.2 V, corresponding to dihydroxyphenyl moieties) decreased their intensity with respect to extracts under milder conditions; conversely, the least labile ones (peak at ca. 0.6 V) remained practically constant even under strong experimental conditions.

In a general comparison of our results with other recent studies published in the literature, Musolino et al. also reported that oleuropein and luteolin-7-O-glucoside are the two main polyphenolic compounds in olive leaf extract [32]. Beyond the exhaustive characterization of olive leaf extracts obtained with conventional and eutectic solvents by Mir-Cerdà et al. [26], other authors explored various types of waste generated in the production of olive oil, including leaves, to evaluate their possibilities as a source of phenolic and terpenic compounds [33].

As of now, NADES have scarcely been used to recover phenolics from this waste type [18,22,24,26,34]. Beyond the above-commented examples, Mitar and Kardum relied on NADES containing aluminum oxide nanoparticles to facilitate the transfer of polyphenols from olive leaves [34]. Although extraction with NADES is fast and efficient, solvent removal and further extract purification may be an unsolved challenge. In this scenario, resin-based procedures are sometimes applied to isolate the bioactive compounds from the solvent and obtain enriched by-products [35]. Conversely, most authors preferred traditional solvents, such as methanol, ethanol, and water, or their mixtures [19,20,21,36], also obtaining good performances regarding the recovery of polyphenols or overall antioxidant capacity; additionally, EtOH/water mixtures also guaranteed the greenness of the method [36]. For instance, another study compared the extraction ability of MeOH and water, demonstrating that results were better when working with hydroalcoholic mixtures, especially when trying to extract some less polar compounds such as oleuropein [37].

Regarding extraction techniques, traditional options, including maceration or decoction, have an evident green nature, although they are methodologically slow [38]. In this context, homogenization-assisted extraction, UAE, and MAE seem to be very popular among the scientific community; anyway, other innovative options, such as pulsed electric fields and high-pressure extraction, also demonstrate high performance, becoming highly competitive in terms of cost and scaling possibilities [26,39,40].

**Figure 6:**
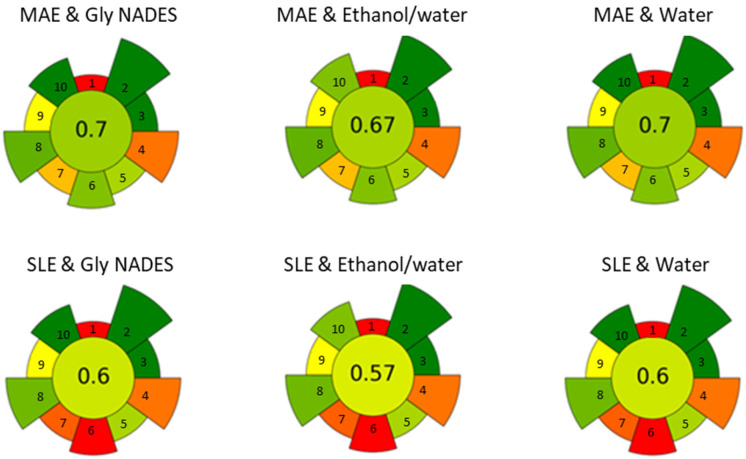
Evaluation of the greenness and sustainability of the sample treatment using the AGREE approach.

Finally, a greenness assessment of the different approaches was conducted to evaluate the sustainability of the extraction process. The AGREEprep calculator [28], which assigns a so-called normalized AGREE score based on the principles of green chemistry, was used. The score ranges from 0 to 1, with values closer to 1 indicating a greener process. Both MAE and SLE techniques were evaluated using water, 20% ethanol, and ChCl/Gly as the solvents. For all solvents tested, the AGREE score for MAE was consistently higher (0.74, 0.71, 0.74) compared to the corresponding SLE system (0.63, 0.60, 0.63) (see Figure 6). The main reason for the difference in scores between the two extraction techniques is the sample throughput, with MAE being more versatile than SLE. The lower scores observed for the ethanol/water system, whether using MAE or SLE, are mostly linked to operator safety concerns. This assessment was performed on a laboratory scale, and factors like sample throughput may differ when considering upscaling.

In summary, ChCl/Gly NADES is the best option considering antioxidant activity and extraction of individual bioactive compounds. While MAE (80 °C, 10 min) provides slightly better recovery than SLE (80 °C, 2 h), along with a higher AGREE score and shorter processing time, SLE remains interesting for large-scale applications due to its simplicity and ease of scaling.

## 4. Conclusions

This study compared various green solvents, including NADES, ethanol/water mixtures, and pure water, for recovering antioxidant compounds from olive leaf waste generated during extra virgin oil production. The performance of SLE was evaluated via mechanical stirring and MAE, analyzing the extracts using FRAP antioxidant capacity, HPLC, and voltammetry. The optimization of working conditions yielded comparable results for most solvents in terms of polyphenolic content and antioxidant capacity. This equivalence is probably due to achieving near-quantitative extraction, limiting further improvements. It should be noted, however, that when less efficient solvents are used (in the case of water or the ethanol/water mixture), the working conditions to achieve similar performances become more extreme, requiring longer times or higher temperatures.

The MAE/NADES combination demonstrated high efficiency with short process times (10 min) and reasonable temperatures (80 °C). Among the NADES tested, choline chloride-glycerol was the most effective, matching the urea-based NADES, without degradation or unpleasant odors. Nonetheless, while MAE/NADES being promising, scalability considerations may impact its cost-effectiveness. SLE using water or ethanol/water mixtures provided excellent results at 80 °C but with longer processing times (120 min). Combining SLE with an ethanol/water mixture appears practical for large-scale operations, but its AGREEprep score is lower compared to MAE options.

For industrial applications, balancing efficiency, scalability, cost, and sustainability is crucial. Nonetheless, developing sustainable and efficient processes for valorizing olive leaf waste will require pilot-scale studies to validate laboratory results, as well as investigating solvent recycling to enhance sustainability.

## Data Availability

All the data is in the paper or in the Supplementary Material.

## Supplementary Materials

The following supporting information can be downloaded at: https://www.mdpi.com/article/10.3390/antiox14020136/s1, Figure S1. Recovery of individual phenolic compounds from olive leaves using water, 20/80 ethanol/water mixture (*v*/*v*) and ChCl/Gly NADES (1:5 m:m, 30% water) using MAE as extraction technique, analyzed by HPLC-UV (mg g^−1^), showing (a) luteolin glucoside, (b) rutin, (c) luteolin, (d) *p*-coumaric acid. Figure S2. MAE study of temperature (80–180 °C) at 10 min of extraction time by FRAP (mg TE g^−1^). Figure S3. SLE study of time (5–120 min) at 80 °C of extraction temperature. Analysis of (a) TPC by HPLC-UV (mg GAE g^−1^), (b) TPC by FRAP (mg TE g^−1^), (c) luteolin glucoside (mg g^−1^), (d) rutin (mg g^−1^), (e) luteolin (mg g^−1^). Figure S4. Influence of NADES composition on the recovery of phenolic compounds, estimated by HPLC-UV, from olive leaves using MAE (blue bars) and SLE (orange bars) as extraction techniques, showing (a) TPC (mg GAE g^−1^), (b) luteolin glucoside (mg g^−1^), (c) rutin (mg g^−1^), (d) luteolin (mg g^−1^). Figure S5. Influence of extraction temperature and time on the recovery of phenolic compounds, estimated by HPLC-UV, from olive leaves using MAE as extraction technique and Gly-based NADES, showing (a) TPC (mg GAE g^−1^), (b) luteolin glucoside (mg g^−1^), (c) rutin (mg g^−1^), (d) luteolin (mg g^−1^). Figure S6. Influence of extraction temperature and time on the recovery of phenolic compounds, estimated by HPLC-UV, from olive leaves using MAE as extraction technique and urea-based NADES, showing (a) TPC (mg GAE g^−1^), (b) luteolin glucoside (mg g^−1^), (c) rutin (mg g^−1^), (d) luteolin (mg g^−1^).
