# Supplementary material for: From Waste to Resource: Exploring Green Approaches for Phenolics Recovery from Olive Leaves
Source: Antioxidants (Basel). 2025 Jan 24;14(2):136. doi: 10.3390/antiox14020136 (PMC11852040; doi:10.3390/antiox14020136)
Supplement: Supplementary file 1 [file antioxidants-14-00136-s001.zip › antioxidants-3362901-supplementary.pdf]

**Supplementary material**

# From Waste to Resource: Exploring Green Approaches for Phenolics Recovery from Olive Leaves

**Paulina Tapia-Quirós [1,2], Aina Mir-Cerdà [1,3], Mercè Granados [1,3], Sonia Sentellas [1,3,4] and Javier Saurina [1,3,*]**

1 Department of Chemical Engineering and Analytical Chemistry, Universitat de Barcelona, Martí i Franquès 1-11, E08028 Barcelona, Spain; paulina.tapia@ub.edu (P.T.-Q.); ainamir@ub.edu (A.M.-C.); mgranados@ub.edu (M.G.); sonia.sentellas@ub.edu (S.S.)

2 Chemical Engineering Department, Escola d'Enginyeria de Barcelona Est (EEBE), Universitat Politècnica de Catalunya (UPC)-BarcelonaTECH, Eduard Maristany 10-14, Campus Diagonal Besòs, E08930 Barcelona, Spain

3 Research Institute in Food Nutrition and Food Safety, Universitat de Barcelona, Av. Prat de la Riba 171, Edifici Recerca (Gaudí), E08921 Santa Coloma de Gramenet, Spain

4 Serra Húnter Fellow, Generalitat de Catalunya, E08003 Barcelona, Spain

* Correspondence: xavi.saurina@ub.edu

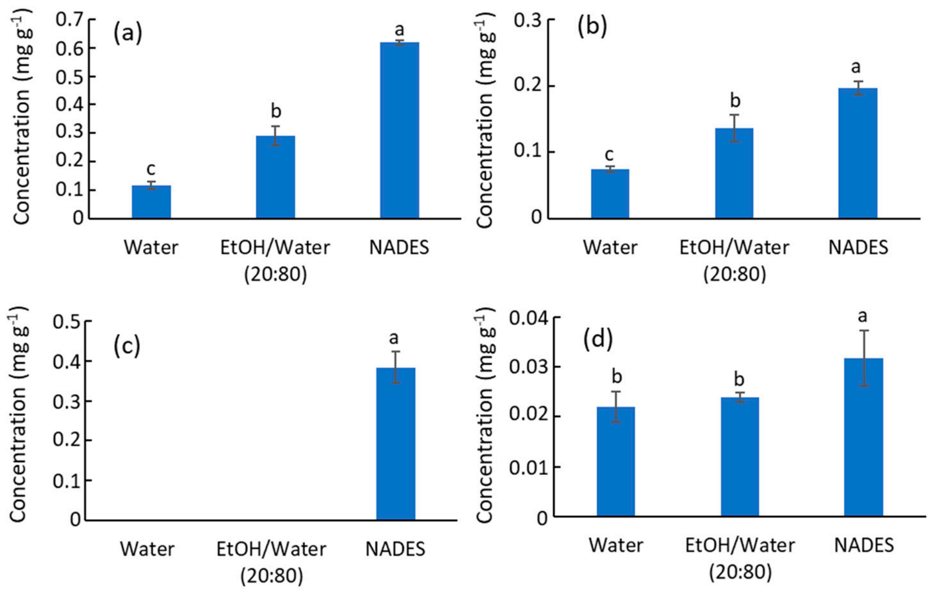


Figure S1. Recovery of individual phenolic compounds from olive leaves using water, 20/80 ethanol/water mixture (v/v) and ChCl/Gly NADES (1:5 m:m, 30% water) using MAE as extraction technique, analyzed by HPLC-UV (mg $g^{-1}$). (a) luteolin glucoside, (b) rutin, (c) luteolin, (d) *p*-coumaric acid. Different letters mean significant differences and vice versa.

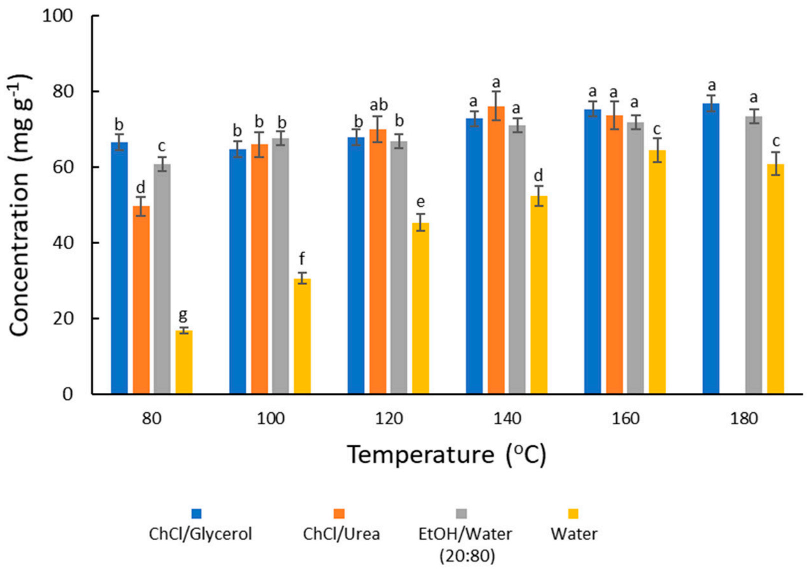


Figure S2. MAE study of temperature (80-180°C) at 10 min of extraction time by FRAP (mg TE $g^{-1}$). Different letters mean significant differences and vice versa.

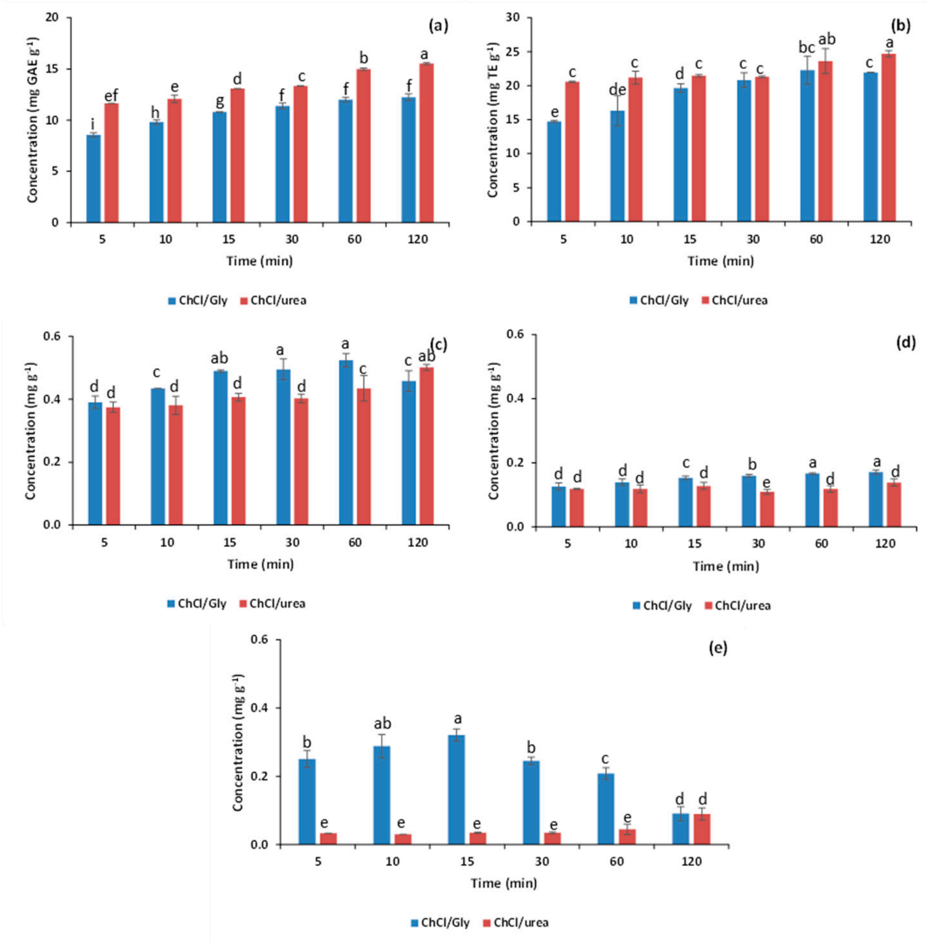


Figure S3. SLE study of time (5-120 min) at 80°C of extraction temperature. Analysis of (a) TPC by HPLC-UV (mg GAE $g^{-1}$), (b) TPC by FRAP (mg TE $g^{-1}$), (c) luteolin glucoside (mg $g^{-1}$), (d) rutin (mg $g^{-1}$), (e) luteolin (mg $g^{-1}$). Different letters mean significant differences and vice versa.

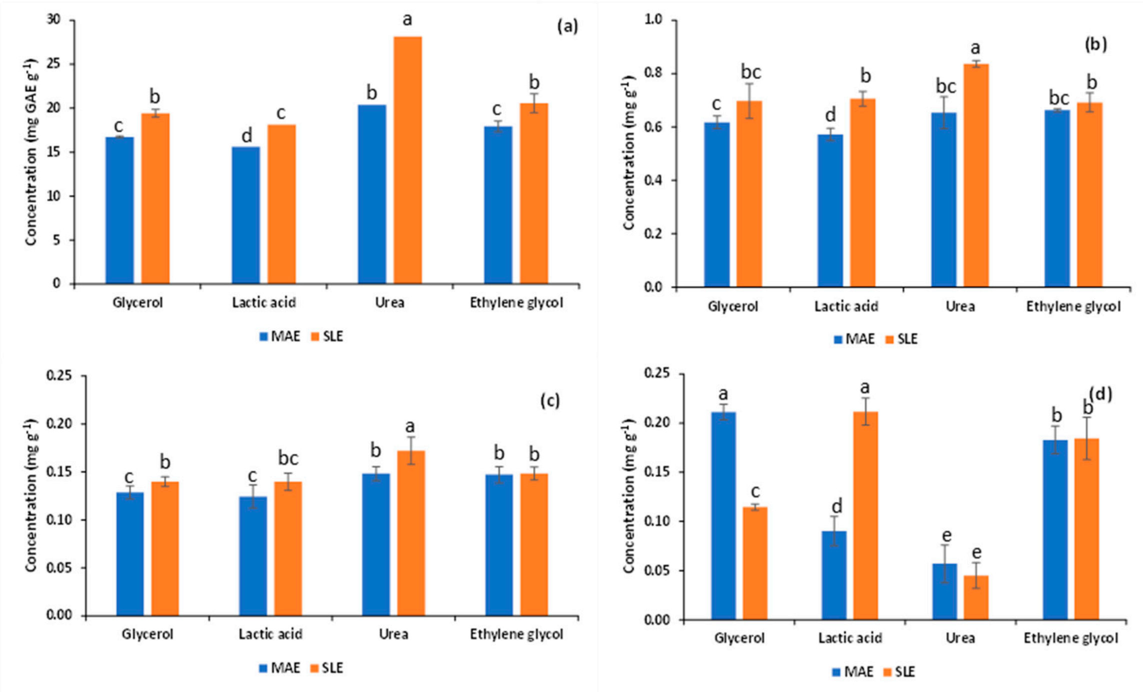


Figure S4. Influence of NADES composition on the recovery of phenolic compounds, estimated by HPLC-UV, from olive leaves using MAE (blue bars) and SLE (orange bars) as extractionve techniques. (a) TPC (mg GAE $g^{-1}$), (b) luteolin glucoside (mg $g^{-1}$), (c) rutin (mg $g^{-1}$), (d) luteolin (mg $g^{-1}$). Different letters mean significant differences and vice versa.

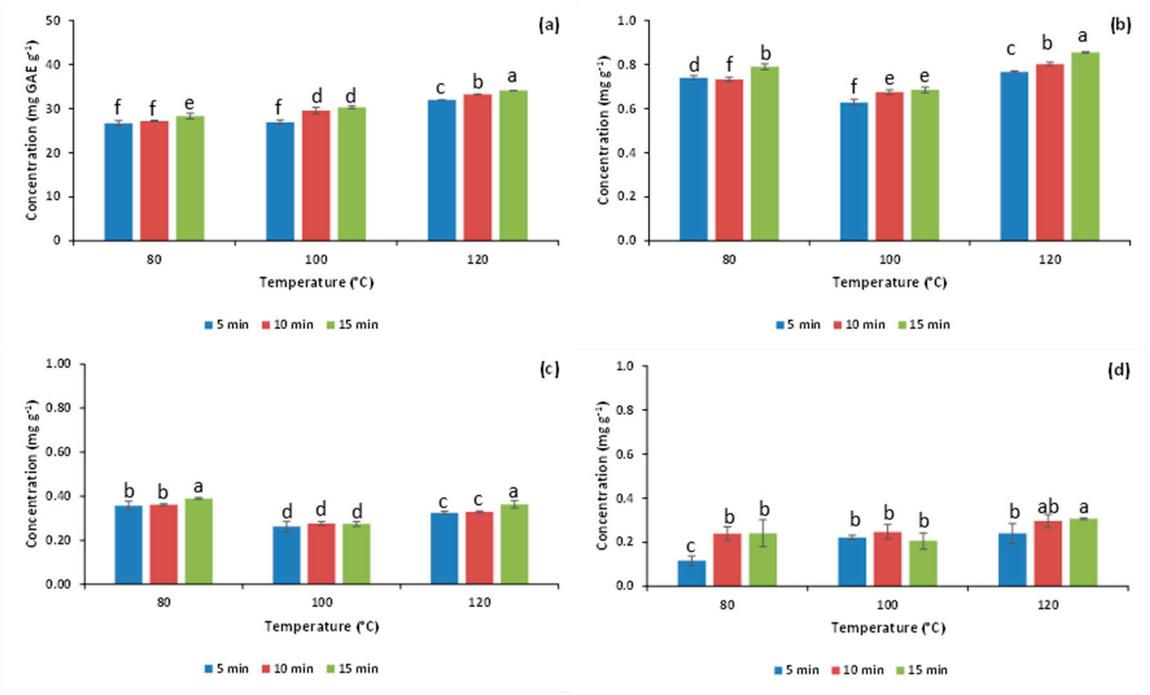


Figure S5. Influence of extraction temperature and time on the recovery of phenolic compounds, estimated by HPLC-UV, from olive leaves using MAE as extraction technique and Gly-based NADES. (a) TPC (mg GAE $g^{-1}$), (b) luteolin glucoside (mg $g^{-1}$), (c) rutin (mg $g^{-1}$), (d) luteolin (mg $g^{-1}$). Different letters mean significant differences and vice versa.

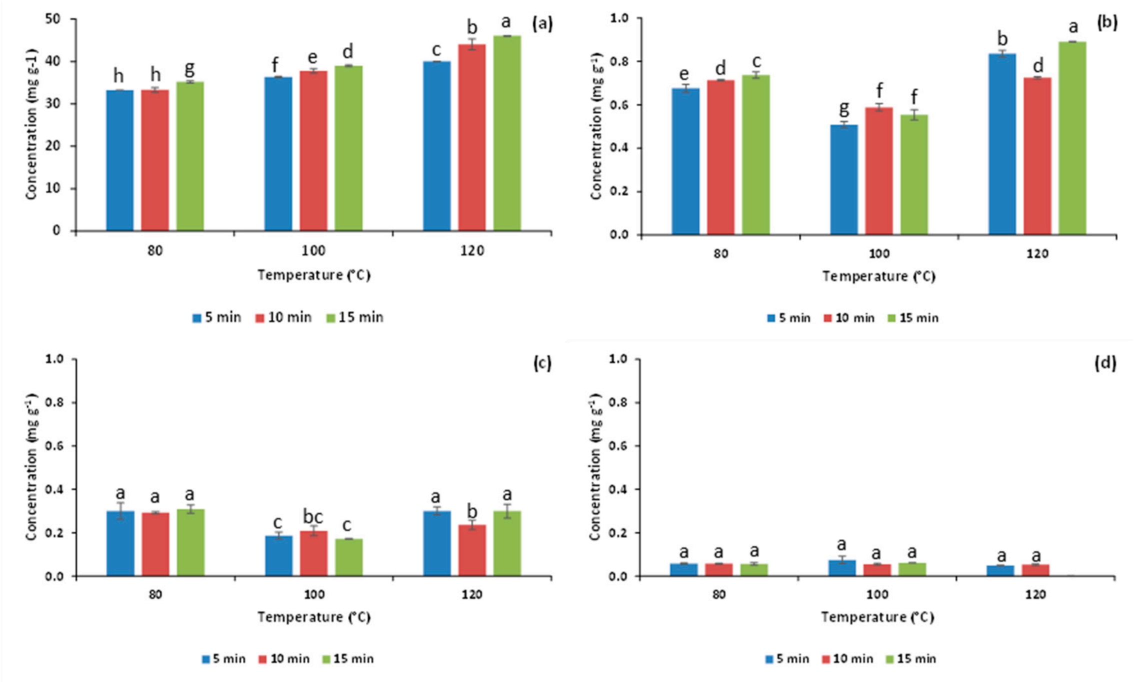


Figure S6. Influence of extraction temperature and time on the recovery of phenolic compounds, estimated by HPLC-UV, from olive leaves using MAE as extraction technique and urea-based NADES. (a) TPC (mg GAE $g^{-1}$), (b) luteolin glucoside (mg $g^{-1}$), (c) rutin (mg $g^{-1}$), (d) luteolin (mg $g^{-1}$). Different letters mean significant differences and vice versa.
